# NX210c drug candidate peptide strengthens mouse and human blood-brain barriers

**DOI:** 10.1186/s12987-024-00577-x

**Published:** 2024-09-27

**Authors:** Chris Greene, Nicolas Rebergue, Gwen Fewell, Damir Janigro, Yann Godfrin, Matthew Campbell, Sighild Lemarchant

**Affiliations:** 1 Smurfit Institute of Genetics, Trinity College Dublin, Lincoln Place Gate, Dublin 2, Ireland; 2 Axoltis Pharma, 60 avenue Rockefeller, Lyon, 69008 France; 3 SynVivo, Huntsville, AL 35806 USA; 4 Flocel Inc., Cleveland, OH 44103 USA; 5 Godfrin Life-Sciences, Caluire-et-Cuire, 69300 France

**Keywords:** BBB leakage, BBB repair, SCO-spondin-derived peptide, Treatment

## Abstract

**Background:**

Alterations of blood-brain barrier (BBB) and blood-spinal cord barrier have been documented in various animal models of neurodegenerative diseases and in patients. Correlations of these alterations with functional deficits suggest that repairing barriers integrity may represent a disease-modifying approach to prevent neuroinflammation and neurodegeneration induced by the extravasation of blood components into the parenchyma. Here, we screened the effect of a subcommissural organ-spondin-derived peptide (NX210c), known to promote functional recovery in several models of neurological disorders, on BBB integrity in vitro and in vivo.

**Methods:**

In vitro, bEnd.3 endothelial cell (EC) monolayers and two different primary human BBB models containing EC, astrocytes and pericytes, in static and microfluidic conditions, were treated with NX210c (1-100 µM), or its vehicle, for 4 h and up to 5 days. Tight junction (TJ) protein levels, permeability to dextrans and transendothelial electrical resistance (TEER) were evaluated. In vivo, young and old mice (3- and 21-month-old, respectively) were treated daily intraperitoneally with NX210c at 10 mg/kg or its vehicle for 5 days and their brains collected at day 6 to measure TJ protein levels by immunohistochemistry.

**Results:**

NX210c induced an increase in claudin-5 protein expression after 24-h and 72-h treatments in mouse EC. Occludin level was also increased after a 24-h treatment. Accordingly, NX210c decreased by half the permeability of EC to a 40-kDa FITC-dextran and increased TEER. In the human static BBB model, NX210c increased by ∼ 25% the TEER from 3 to 5 days. NX210c also increased TEER in the human 3D dynamic BBB model after 4 h, which was associated with a reduced permeability to a 4-kDa FITC-dextran. In line with in vitro results, after only 5 days of daily treatments in mice, NX210c restored aging-induced reduction of claudin-5 and occludin levels in the hippocampus, and also in the cortex for occludin.

**Conclusions:**

In summary, we have gathered preclinical data showing the capacity of NX210c to strengthen BBB integrity. Through this property, NX210c holds great promises of being a disease-modifying treatment for several neurological disorders with high unmet medical needs.

**Supplementary Information:**

The online version contains supplementary material available at 10.1186/s12987-024-00577-x.

## Background

The blood-brain barrier (BBB) is a gatekeeper of the brain [1]. Altogether, endothelial cells, pericytes and astrocyte endfeet carefully regulate the passage of nutrients, molecules and cells between the blood and the brain [2]. When tight junction (TJ) proteins attaching endothelial cells to each other are disrupted, the BBB is readily interrupted and leaks blood-derived proteins, such as fibrinogen and albumin into the brain, causing a vicious circle of neuroinflammatory and neurodegenerative processes [2]. A growing body of evidence indicates that BBB disruption/leakage may be a major contributor of the pathogenesis and/or progression of neurovascular and neurotraumatic events [3], but also less expectedly of psychiatric [4–7], autoimmune [8–10], or neurodegenerative diseases, including amyotrophic lateral sclerosis (ALS) [11–16], Alzheimer’s disease (AD) [17, 18], Parkinson’s disease (PD) [19, 20], or Huntington disease [21–23]. Associations of BBB dysfunction/leakage and clinical deficits related to affected brain regions are emerging in the literature and show that elevated BBB leakage is a predictor of higher disease severity and worse clinical disability [5, 7, 11, 24–32]. Most current treatments for the neurodegenerative diseases listed above are designed to tackle inflammation or neurodegeneration rather than restoring BBB integrity. Safeguarding BBB integrity is a promising disease-modifying therapeutic strategy for patients suffering from neurological diseases/injuries with high unmet medical needs where the barrier is dysfunctional.

NX210c is a 12-amino acid peptide designed from the most conserved sequence of the type 1 thrombospondin repeats (TSR1) of the subcommissural organ (SCO)-spondin, a central nervous system (CNS)-specific glycoprotein involved in neurogenesis and neuronal development during embryogenesis [33]. Recently, truncated variants of the SCO-spondin were detected in patients with a schizoaffective disorder, and a knock-in mouse model for these variants presented with BBB disruption [34]. We have previously demonstrated that NX210c reduced glutamate-induced excitotoxicity in cortical and hippocampal neurons of rat and human origins in vitro by promoting phosphoinoside-3-kinase/mammalian target of rapamycin (PI3K/mTOR) survival pathway and reducing apoptosis, a neuroprotective mechanism mediated by integrins containing the β_1_ subunit [35]. In addition to saving neurons, NX210c also enhanced glutamatergic receptor post-synaptic currents which promoted synaptic transmission in the hippocampus in basal conditions [36], or restored it in hypoxic conditions [37]. To better treat patients suffering from neurological disorders, it is necessary both to treat neurons and to restore the impermeability of the BBB which would prevent the entry of toxic and inflammatory blood components into the brain as described above.

The aim of this study was to evaluate if NX210c could reinforce BBB integrity. We first screened its modulatory effect on the expression of TJ proteins (claudin-5, occludin, zonula occludens-1 (ZO-1)), and on barriers integrity/permeability using bEnd.3 endothelial cell monolayers, and primary human static and dynamic BBB models. Remarkably, NX210c peptide increased the expression of claudin-5 and occludin, and favorably modulated the integrity and permeability of BBBs, including in the most complex model used (i.e., primary human microfluidic model exhibiting endothelial cells, astrocytes and pericytes). In vivo, intraperitoneal injections of the peptide once a day for 5 days to old mice increased the protein expression of claudin-5 and occludin in the hippocampus, and in the cortex too for occludin. By treating the neurovascular unit as a whole, NX210c may represent a first-in-class disease-modifying treatment for neurological disorders for which no satisfactory solutions exist so far.

## Methods

### NX210c peptide synthesis

NX210c is the oxidized cyclic form of a linear dodecapeptide derived from the most conserved TSR1 consensus sequence of the SCO-spondin. NX210c sequence is H-WSGWSS[CSRSC]G-OH; the brackets represent the disulfide bond between cysteine residues. It was manufactured by GENEPEP (Saint Jean-de-Védas, France) and supplied as an acetate salt lyophilizate whose purity was assessed as 96% using high-performance liquid chromatography. NX210c was dissolved in cell culture water or water for injection for in vitro and in vivo experiments, respectively.

### Mouse endothelial cell monolayers

#### Cells

Mouse brain endothelial cell line (bEnd.3; CRL-2299, American Type Culture Collection, Manassas, VA, USA) was cultured in Dulbecco’s Modified Eagle’s Medium (DMEM) containing 2 mM sodium pyruvate (31966021, ThermoFisher Scientific, Waltham, MA, USA) supplemented with 10% fetal bovine serum (FBS; F7524, Merck Millipore, Burlington, MA, USA) in a 5% CO_2_ incubator at 37 °C.

For permeability and transendothelial electrical resistance (TEER) experiments, 5 × 10^4^ cells were seeded on 6.5-mm diameter, 0.4-µm pore, polyester membrane HTS transwell^®^ inserts (3378, Corning Star, Cambridge, MA, USA), which corresponds to ∼ 1.5 × 10^5^ cells/cm^2^, on 24-well plates. Inserts were coated beforehand with 50 µg/mL fibronectin (F1141, Merck Millipore) for 1 h at 37 °C and then washed three times with phosphate buffered saline (PBS; 14190, Biosciences, Dublin, Ireland).

For immunocytochemistry experiments, 1 × 10^5^ cells were seeded on Nunc Lab-Tek II Chamber Slides (154534PK, ThermoFisher Scientific), which corresponds to ∼ 1.4 × 10^5^ cells/cm^2^. Lab-teks were coated beforehand with 50 µg/mL fibronectin for 1 h at 37 °C and then washed three times with PBS.

For western-blot experiments, 2.5 × 10^5^ cells per well were seeded on 12-well plates (83.3921, Sarstedt, Nümbrecht, Germany), which corresponds to ∼ 7 × 10^4^ cells/cm^2^.

#### NX210c treatment

Two days after seeding, endothelial cell monolayers were treated with NX210c at 1, 10 and 100 µM or its vehicle (= NX210c vehicle; BE17-724, Lonza, Basel, Switzerland), for 24 h and 72 h, to perform immunocytochemistry of claudin-5 at TJs, and TEER and permeability assays. Parallel cultures were treated with cell culture water (= NX210c vehicle) or NX210c at 1, 10 and 100 µM, for 1 h, 3 h, 6 h, 24 h, 48 h and 72 h, and subsequently messenger ribonucleic acids (mRNAs) and proteins were extracted for reverse transcription quantitative polymerase chain reaction (RT-qPCR) and western-blot analyses, respectively, to assess the expression of claudin-5, ZO-1 and occludin (most part of these experiments are presented in the Supplementary materials).

#### TEER assay

Prior to measurements, the media in apical and basolateral chambers was replaced with fresh medium, and the devices were allowed to equilibrate to room temperature (RT) for 10 min. TEER of mouse endothelial cell monolayers was then monitored using an EVOM resistance meter (EVOM2, Merck Millipore) fitted with chopstick-like electrodes. TEER values (ohm × cm^2^) were normalized by subtracting TEER values without cells (blank). The results are expressed as percentages of the control group.

#### Permeability assay

The culture media in the apical chamber was replaced with fresh media containing 1 mg/mL of a 40-kDa fluorescein isothiocyanate (FITC)-dextran (FD40, Sigma-Aldrich, Saint-Louis, MO, USA) filtered with a 0.22-µm filter beforehand. Sampling aliquots were taken from the basolateral chamber and replaced with fresh medium every 15 min for 2 h. FITC-dextran fluorescence was determined using a spectrofluorometer (FLUOstar OPTIMA, Optima Scientific, Parklands, South Africa) at an excitation wavelength of 485 nm and an emission wavelength of 520 nm. Relative fluorescence units were converted to values of nanograms per milliliter, using FITC-dextran standard curves, and were corrected for background fluorescence and serial dilutions over the course of the experiment. The apparent permeability coefficient (Papp) for each treatment was calculated using the following equation, as previously described [38]: Papp (cm/s) = (dQ / dT)(A × C0) where dQ / dT (µg/s) is the rate of appearance of FITC-dextran in the basolateral chamber after application, A (cm^2^) is the effective surface area of the insert size, and C0 (µg/mL) is the initial FITC-dextran concentration on the donor side. dQ / dT is the slope m (y = mx + c) calculated by plotting the cumulative amount (Q) versus time (s). The results are expressed as percentages of the control group.

#### Immunocytochemistry

Cells were fixed for 10 min at RT with 4% paraformaldehyde (PFA; P6148, Merck Millipore) in PBS, washed twice with PBS and incubated with blocking buffer containing 5% normal goat serum (31872, ThermoFisher Scientific) and 0.05% Triton X-100 (T8787, Merck Millipore) in PBS for 1 h at RT before overnight incubation with polyclonal rabbit anti-claudin-5 (34-1600 [39], 1:100 dilution; Invitrogen, Carlsbad, CA, USA) in blocking buffer at 4 °C. Cells were then washed three times with PBS and incubated with goat anti-rabbit Cy3 secondary antibody (ab6939, dilution 1:500; Abcam, Cambridge, UK) in blocking buffer for 2 h at RT and counterstained with Hoechst 33,258 (94403, Merck Millipore) to visualize nuclei. Slides were removed from the chamber and mounted with Aqua Poly/Mount (18606, Polysciences, Warrington, PA, USA) before visualization using a confocal laser scanning /microscopy (LSM 710, Zeiss, Oberkochen, Germany). Images were taken at 20× magnification (dry objective with 0.8 numerical aperture) and converted to 8-bit. The Despeckle function was used to remove noise and images were converted to binary and the percentage immunoreactive area was measured using ImageJ software at predefined range (512 × 512 pixels, 40 threshold). The results are expressed as percentages of the control group.

#### Western-blot

After mechanical dissociation using a lysis buffer containing 62.5 mM Tris (T6066, Sigma-Aldrich), 2% sodium dodecyl sulfate (SDS; L3771, Sigma-Aldrich), 10 mM dithiothreitol (D0632, Sigma-Aldrich) and one cOmplete™, Mini Protease Inhibitor Cocktail (11836153001, Sigma-Aldrich) / 10 mL, cell layers were centrifuged at 12,000 g for 20 min at 4 °C, and protein concentrations of supernatants were measured using the Pierce BCA protein assay (23225, ThermoFisher Scientific). Ten (10) µg total protein were loaded per well for separation by SDS-polyacrylamide gel electrophoresis (12% acrylamide). Gels were transferred onto methanol-activated polyvinylidene difluoride membranes (IPVH00010, Merck Millipore) via semi-dry transfer. Transferred membranes were reactivated with methanol (Trinity College Dublin, Ireland) and blocked under slight agitation in tris-buffered saline (TBS)- Tween-20 (0.1%) containing 3% weight/volume Marvel non-fat dry milk for 1 h at RT. Blocked membranes were washed three times with TBS-Tween-20 and probed with primary rabbit polyclonal antibodies targeting claudin-5 (34–100, 1:1,000 dilution; Invitrogen), occludin (NBP1-87402 [40], 1:1,000 dilution; Novus Biotech, Centennial, CO, USA), ZO-1 (402200 [41, 42], 1:1,000 dilution; Invitrogen) or β-actin (ab8227, 1:4,000 dilution; Abcam) in blocking buffer overnight at 4 °C. Membranes were then washed three times for 5 min in TBS-Tween-20 and incubated with horse radish peroxidase-conjugated goat anti-rabbit secondary antibody (A6154; Sigma-Aldrich) diluted 1:2,000 in TBS-Tween-20 for 2 h at RT. Membranes were washed four times for 5 min in TBS-Tween-20. Proteins were visualized using WesternBright ECL HRP substrate kit (K-12045-D50, Advansta, San Jose, CA, USA), and images were acquired using C-Digit^®^ Blot Scanner (LICORbio™, Lincoln, NE, USA). Densitometry was performed using ImageJ with protein bands of interest normalized to the loading control β-actin. The results are expressed as percentages of the control group.

### Human static BBB triculture model

#### Cells

Human brain microvascular endothelial cells (HBMVEC; ACBRI-376, Cell Systems, Kirkland, WA, USA) [43] were grown in T75 flasks in endothelial basal medium (EBM)-2 (CC-3162, Lonza) containing 5% FBS (S12450H, R&D Systems, Minneapolis, MN, USA) and CultureBoost™ (4CB-500, Cell systems), a broad-spectrum mitogen, at a concentration of 50 µg/mL. Normal human astrocytes (NHA; ACBRI-371, Cell Systems) [43] were grown in T75 flasks in DMEM (30-2002, American Type Culture Collection) containing 1X B27 (A1486701, Gibco, ThermoFisher Scientific), 5% FBS, 1X Non-Essential Amino Acids (NEAA) (100X) (11-140-050, Gibco, ThermoFisher Scientific) solution, 1X Antibiotic-Antimycotic (15-240-096, Gibco, ThermoFisher Scientific) and 1X glutamate (AAJ6057314, ThermoFisher Scientific). Human brain vascular pericytes (HBVP; 1200, ScienCell, Carlsbad, CA, USA) were grown in T75 flasks in DMEM containing 5% FBS, 1X NEAA, 1X Antibiotic-Antimycotic and 1X glutamate.

For each cell type, cells were transferred into a 10-mL conical tube (430791, Corning, New York, NY, USA) containing 2 mL of their respective culture medium, before being centrifuged at 313 g for 5 min at RT. Supernatants were removed and cells resuspended in 5 mL of culture medium before seeding them into poly-L-lysine-coated T75 flasks (Greiner Bio-One 658930, ThermoFisher Scientific) at 5 × 10^3^ cells/cm^2^. Flasks were incubated at 37 °C with 5% CO_2_. The media was changed whenever needed until cells reach ∼ 90–95% confluence. Cells were then rinsed twice with Dulbecco’s phosphate-buffered saline (DPBS; 02-0019-0500, VWR, Radnor, PA, USA) before adding 3 mL of 0.25% trypsin/ethylenediaminetetraacetic acid (T/E) solution (25-053-CI, Corning) in the flask for 2–3 min. 5 mL of culture medium was added to the cells treated with T/E solution before centrifugation at 313 g for 5 min at RT. The cells were resuspended in culture medium before seeding them to new poly-L-lysine-coated flasks at the same cell density as described above. The flasks were incubated at 37 °C with 5% CO_2_. The media was changed whenever needed until cells reach ∼ 90–95% confluence.

Polyester membrane transwell^®^ inserts (0.4-µm pore, 6.5-mm diameter; EF5650T.3470, Corning Star) in 24-well plates were coated with 10 µL of Cell Systems Corporation Attachment Factor (4Z0-210, ThermoFisher Scientific) for 1 h at RT to allow optimal cell attachment and growth [44]. Thirty-five (35) × 10^3^ endothelial cells were seeded in the apical chamber (which corresponds to ∼ 1.05 × 10^5^ cells/cm^2^), and 40 × 10^3^ astrocytes and 40 × 10^3^ pericytes in astrocyte/pericyte mixed culture medium (ratio 1:1) in the basolateral chamber (which corresponds to ∼ 2.1 × 10^4^ cells/cm^2^ in total). Cultures were considered differentiated when astrocytic feet extend to the endothelial cell monolayer. At ∼ 80–90% confluency, endothelial cells were grown without mitogen for 2 days.

#### NX210c treatment

Endothelial cells were treated with NX210c at 10 and 100 µM, or its vehicle (= cell culture water, 10977-015, Invitrogen), for 24 h and up to 5 days to perform TEER assay.

#### TEER assay

Prior to measurements, the media in apical and basolateral chambers was replaced with fresh medium, and the devices were allowed to equilibrate to RT for 10 min. TEER was then monitored using a Millicell-ERS (Electrical Resistance System) Volt-Ohm meter (MERS00001, Merck Millipore) connected to the EndOhm chamber (ENDOHM-6G, World Precision Instruments, Sarasota, FL, USA). TEER values (ohm × cm^2^) were normalized by subtracting TEER values without cells (blank). The results are expressed as percentages of the control group.

### Human dynamic BBB triculture model

#### Chips

Each chip (102005-3, SynVivo, Huntsville, AL, USA) contains two independent vascular channels (apical chambers) containing human primary brain microvascular endothelial cells with flow access openings surrounding the tissue compartment (basolateral chamber) containing human primary cortical astrocytes and brain vascular pericytes in the center of the chip. Of note, only one apical chamber and the basolateral chamber was used in this experiment. The dimensions of the apical chamber were 200-µm width × 100-µm height, and the dimensions of the basolateral chamber were 1.8-mm diameter × 100-µm height. The apical chambers are in communication with the basolateral chamber through a series of porous interfaces of 3-µm width × 3-µm height × 100-µm length dimensions spaced every 50 μm apart over a 2550-µm arc length along the apical chamber. The chip is assembled on a microscope glass slide with plastic tubes allowing access to the apical and basolateral chambers individually. Ports A, C, and E serve as inlets and ports B, D, and F as outlets of culture media with or without cells for apical (A, B, E, F) and basolateral (C, D) chambers. For IMN2 Radial-TEER chips, the chips are equipped by electrode ports, one for each apical and basolateral chamber. Three days prior to cell seeding in microfluidic devices, chips were injected with 200 µg/mL human fibronectin (FC010, Sigma-Aldrich) in PBS, and then attached to the pneumatic primer device (205001, SynVivo) and primed at 7 psi for 15 min at RT. The chips were then incubated for 1 h at 37 °C with 5% CO_2_. Chips were then perfused fully with endothelial cell growth medium (ECGM; MD-0010B, iXCells Biotechnologies, San Diego, CA).

#### Cells

HBMVECs (10HU-051, iXCells Biotechnologies) were resuspended in ECGM containing endothelial cell growth supplement (MD-0010, iXCells Biotechnologies). Human cortical astrocytes (Catalog #1800, ScienCell) were resuspended in astrocyte medium containing 2% of FBS, 1% of astrocyte growth supplement and 1% of penicillin/streptomycin (P/S) solution (Catalog #1801, ScienCell). Human brain vascular pericytes (Catalog #1200, ScienCell) were resuspended in pericyte medium containing 2% of FBS, 1% of pericyte growth supplement and 1% of P/S solution (Catalog #1201, ScienCell). T75 flasks (156367, ThermoFisher Scientific) were coated by mixing 15 µL of poly-L-lysine stock (0413, ScienCell) into 10 mL of deionized water and incubating the flasks for 1 h at 37 °C, and then washed with deionized water. For each cell type, 5,000 cells/cm^2^ were seeded separately into poly-L-lysine-coated flasks, and then incubated at 37 °C with 5% CO_2_. The media was changed whenever needed until cells reached ∼ 90% confluence.

Flasks containing HBMVECs were rinsed with PBS and then incubated with 0.05% T/E (24300-054, Gibco, ThermoFisher Scientific) for 3 min at 37 °C for cell detachment. Cells were resuspended in ECGM and centrifuged at 0.2 g for 5 min at RT. The supernatant was discarded, and cells were resuspended at 3 × 10^7^ cells/mL. HBMVECs were injected into the apical chamber of chips at 5 µL/min for 5 s (one side only) with a 1-mL syringe (309628, BD, Franklin Lakes, NJ, USA) using a programmable syringe pump (Harvard Apparatus, Holliston, MA, USA), which corresponds to 12,500 cells or ∼ 81.7 × 10^6^ cells/cm^2^. The chips were then placed into the incubator at 37 °C with 5% CO_2_ for 4 h for cell adhesion. After this time, ECGM was injected in the apical chamber at a flow rate ramp of 0.02 to 0.05 µL/min (which corresponds to 7.58E-03 to 1.92E-02 dyn/cm^2^) for 16 h, and then a constant rate of 0.05 µL/min (which corresponds to 1.92E-02 dyn/cm^2^) for 8 h. The confluence and elongation of endothelial cells was confirmed using an inverted Nikon Eclipse T2 microscope (#600334, Delta Optical Instruments Inc., North Little Rock, AR, USA) combined with Teledyne Photometrics IRIS 15 (AD19D631009, Teledyne Photometrics, Tucson, AZ, USA) prior to the seeding of astrocytes and pericytes.

Flasks containing astrocytes or pericytes were rinsed with 4-(2-Hydroxyethyl)-1-piperazine ethanesulfonic acid (HEPES) buffered saline (CC-5024, Lonza) and then incubated with 0.025% T/E (25200-056, Gibco, ThermoFisher Scientific), diluted in PBS, for 3–5 min at 37 °C for cell detachment. For each cell type, cells were transferred to a 50-mL conical tube (21008-178, Avantor, Radnor Township, PA, USA) containing 5 mL of FBS and 5 mL of astrocyte or pericyte culture media, and then centrifuged at 0.2 g for 5 min at RT. For each cell type, supernatants were discarded, and cells resuspended at 1 × 10^7^ cells/mL. Finally, astrocytes and pericytes were mixed at a 2:1 ratio in their respective culture media (corresponding to 200 µL for astrocytes and 100 µL for pericytes in their respective culture media). Mixed cells were then injected into the basolateral chamber of chips at 5 µL/min for 10 s with a 1-mL syringe using the programmable syringe pump, which corresponds to ∼ 8,300 cells in total, which corresponds to ∼ 3.28 × 10^7^ cells/cm^2^. The chips were then placed into the incubator at 37 °C with 5% CO_2_ for cell adhesion during 3 h. After this time, ECGM was injected in the apical chamber at a flow rate ramp of 0.05 to 0.1 µL/min (which corresponds to 1.92E-02 dyn/cm^2^ to 3.79E-02 dyn/cm^2^) for 16 h, and then a constant rate of 0.1 µL/min (which corresponds to 3.79E-02 dyn/cm^2^) for 32 h. The formation of a 3D lumen on the walls of the apical chamber was confirmed using microscopy prior to experiments.

#### NX210c treatment

Three days after seeding, ECGM containing NX210c at 10 or 100 µM, or its vehicle (= cell culture water, SH30529.02, Dutscher, Bernolsheim, France) was filled in the inlet tube of the apical chamber and injected at a constant rate of 0.1 µL/min for 4 h to perform TEER and permeability assays.

#### TEER assay

Prior to each measurement, the devices were allowed to equilibrate at RT for 10 min. TEER was monitored using a Cell Impedance Analyzer (304001, SynVivo) connected to two silver chloride electrodes, prior to HBMVECs seeding (blank), 5 min prior to treatment (baseline) which corresponds to 72 h post-HBMVECs seeding, and after 4 h of treatment on the same chip. TEER values (kOhms) were normalized by subtracting TEER values without cells (blank). The results are normalized to the baseline 72 h post-seeding (Resistance 4 h – Resistance 72 h post-seeding) and expressed as percentage of the control group.

#### Permeability assay

The culture media in the apical chamber was replaced with fresh serum-free media containing 0.25 mg/mL of a non-filtered 4-kDa FITC-dextran (46944, Sigma-Aldrich) at 0.1 µL/min. This dynamic model offers a better maturation of endothelial cells than those in static models, due to the shear stress forces applied, thereby creating a tighter barrier. Consequently, a 4-kDa FITC-dextran was used in this model instead of the 40-kDa FITC-dextran used in the static model [45].

After 1 h, fluorescent images were acquired at a 4× magnification (3 × 1 frame area) for 200 ms exposure using the Nikon microscope and Nikon Elements software. Nikon Elements software was used to create a region of interest (ROI) in the apical chamber, and the averaged fluorescence intensity of the FITC-dextran within the ROI was measured at endpoint. The same ROI size was used to measure the averaged fluorescence intensity of the FITC-dextran in the basolateral chamber at endpoint. Permeability values were calculated as the ratio of fluorescence intensity between the basolateral and apical chambers. The results are expressed as percentages of the control group.

### In vivo proof of concept study

#### Animals

All experimental procedures were conducted in strict adherence to the European Directive of 22 September 2010 (2010/63/UE) and approved by the French Ministry of Research (#37745_2022062019006001). Eighteen old C57BL/6J littermate male mice (85–88 weeks old, Janvier labs, Le Genest-Saint-Isle, France) were used in this study. Six young C57BL/6J male mice (11–14 weeks old; Janvier labs) from the same breeding colony as old mice were used as an experimental control of a tight barrier. Mice were housed by groups of three per cage in a controlled environment (temperature: 22 ± 2 °C, 12 h / 12 h light/dark cycle, with lights off at 7 p.m.) with free access to food and water. Mice were acclimatized for at least 8 days in the animal facility prior to experiments.

#### Treatment groups

Old mice were randomly assigned to one of two treatment conditions: NX210c or its vehicle (= water for injection; B. Braun, Melsungen, Germany). Six young mice and nine old mice were injected intraperitoneally (i.p.) once a day with vehicle from day 1 (D1) to D5. Nine old mice were injected i.p. once a day with NX210c at 10 mg/kg (dosage volume: 10 mL/kg) from D1 to D5. Operators were blinded to treatment groups. One mouse from the vehicle-treated old mice died before the end of the experiment.

#### Immunohistochemistry

On the next day after the last injection (D6), mice were sacrificed and brains collected to perform immunohistochemistry. Analgesia was provided 30 min before sacrifice via a subcutaneous injection of buprenorphine at 0.05 mg/kg (Buprécare; Alcyon, Neuilly-sur-Seine, France) and then mice were anesthetized with an i.p. injection of a mixture composed of xylazine at 12 mg/kg (Rompun 2%; Alcyon) and ketamine at 90 mg/kg (Ketamine 1000; Alcyon) in saline solution (NaCl 0.9%). Once the required level of anesthesia was reached (respiratory arrest, heart rate below 30 beats/min), PBS (P4417, Sigma-Aldrich) and 4% PFA (252549-L, Sigma-Aldrich) were successively perfused into the heart. Following 2 days post-fixation at 4 °C, brains were dehydrated in 70% ethanol and then immersed consecutively in two baths of 90% ethanol, three baths of 100% ethanol and three baths of methylcyclohexane, before being embedded in wax (Tissue-Tek^®^ Paraffin Wax TEK III; 4508, Sakura, Torrance, CA, USA) using an automated tissue processor (Leica, Wetzlar, Germany). Six 5-µm-coronal Sect. (100 μm apart of each other) from each brain were cut on a microtome (Leica RM2255, Microm HM355S, Leica), collected on Superfrost + slides (J1800AMNZ, ThermoFisher Scientific), dried during 1 h at 60 °C, and stored at RT until analysis. Immunohistochemistry was performed using an automated staining system (Discovery XT; Roche, Basel, Switzerland) and associated reaction buffers containing RiboCC (ref 05266297001; for claudin-5 and occludin) or CC1 (ref 06414575001; for collagen IV and ZO-1/claudin-5 co-staining), both from Roche. Sections were washed and then exposed to primary antibodies for 1 h at RT with dilutions as follows: rabbit polyclonal anti-claudin-5 antibody (34–160, dilution 1:100; ThermoFisher Scientific), rabbit monoclonal anti-occludin antibody (ab216327 [46] dilution 1:100; AbCam), goat polyclonal anti-ZO-1 antibody (ab190085 [47], dilution 1:50; AbCam) or goat polyclonal anti-collagen IV antibody (AB769, dilution 1:50; Sigma Aldrich). Claudin-5, occludin and collagen IV antibodies were used separately, whereas a double-staining was done for claudin-5/ZO-1 in order to specifically analyze ZO-1 present on vessels and thus not to analyze ZO-1 expressed by other cell types [48]. Washed sections were then incubated for 1 h at RT with fluorescent Alexa fluor 488-conjugated secondary antibody (A11008, dilution 1:500; Invitrogen) or fluorescent Alexa fluor 647-conjugated secondary antibody (A21447, dilution 1:500; Invitrogen), then washed again, stained with 300 nM DAPI (D21490, Molecular Probe, Eugene, OR, USA) and finally mounted on coverslips using Fluoromount (K024, CliniSciences, Nanterre, France). Negative controls for unspecific binding of the secondary antibodies were conducted in parallel sections following the same procedures described above, except the incubation in primary antibodies. Sections were imaged using 20× magnification on an Axioscan Z1 microscope (Zeiss). Immunoreactive areas for claudin-5, occludin and collagen IV stainings were quantified using QuPath software (version 0.4.3) using a threshold algorithm of the signal intensity in manually-segmented cortex and hippocampus of the six brain sections. For ZO-1, the analysis was performed similarly except that only ZO-1 co-localized with claudin-5 (= vessel-specific) was used to measure the immunoreactive area. The results are expressed as percentages of the young vehicle group.

### Statistical analyses

All values are expressed as mean ± standard deviation (SD). Statistical analyses were performed using GraphPad Prism software package 10.1.2. and using one-way or two-way analysis of variance (ANOVA) followed by Dunnett’s multiple comparisons test for graphs containing groups that all passed the Shapiro-Wilk normality test and Brown-Forsythe test for group variances. Otherwise, the Kruskal-Wallis followed by Dunn’s test was applied. An alpha level of *p* < 0.05 was used to determine the significance in all the statistical tests.

In the in vivo proof of concept study, three significant outliers were identified using a Grubbs test (α = 0.05) for occludin in the vehicle-treated old mice group in the cortex, and in the vehicle-treated young mice group and in the NX210c-treated old mice group in the hippocampus; they were therefore removed from all the analysis (as described elsewhere [49]).

## Results

### NX210c strengthens the integrity of static bEnd3 mouse endothelial cell monolayers and increases claudin-5 and occludin tight junction proteins

**Fig. 1:**
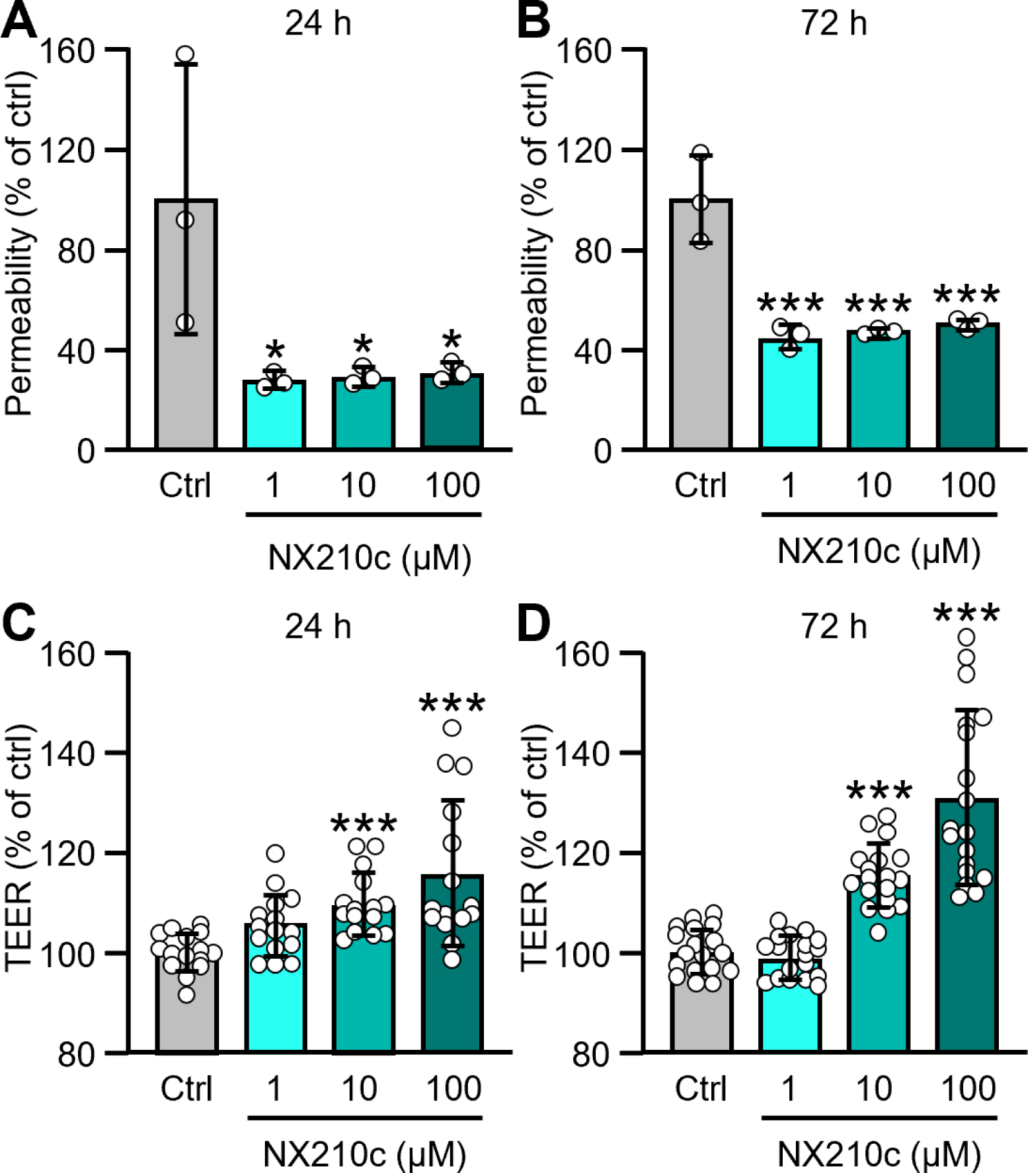
NX210c strengthens the integrity of bEnd3 mouse brain endothelial cell monolayers. (**A-B**) The permeability of bEnd.3 mouse brain endothelial cells grown on transwell^®^ inserts to a 40-kDa FITC-dextran was measured after exposure to NX210c (1, 10, 100 µM) or its vehicle (cell culture water; ctrl) for 24 h (**A**) or 72 h (**B**). One-way ANOVA followed by Dunnett’s multiple comparisons test: ^***^p < 0.0001, ^*^p < 0.05 compared with the control group, *n* = 3 biological replicates/group from one experiment. (**C-D**) In parallel cultures, the TEER was measured using the same experimental design after 24-h (**C**) and 72-h treatments (**D**). Kruskal-Wallis followed by Dunn’s multiple comparisons test: ^***^p < 0.001 compared with the control group, *n* = 15/group from two independent experiments (**C**; i.e., *n* = 6 biological replicates/group from experiment 1 and *n* = 9 biological replicates/group from experiment 2) and *n* = 18/group from two independent experiments (**D**; *n* = 9 biological replicates/group from experiment 1 and *n* = 9 biological replicates/group from experiment 2)

We have first evaluated the permeability of bEnd3 mouse endothelial cell monolayers to a 40-kDa FITC-dextran in transwell^®^ inserts using 3 different concentrations of NX210c (1, 10 and 100 µM) or its vehicle (cell culture water). The FITC-dextran was placed on the apical chamber containing endothelial cells, and the fluorescence was measured 1 h later on the basolateral chamber. A strong reduction of the permeability to the FITC-dextran was observed in presence of NX210c after 24-h treatment (Fig. 1A: -72.5%, -70.9% and − 69.1% for NX210c at 1, 10 and 100 µM compared with control; *p* = 0.0280, *p* = 0.0309 and *p* = 0.0349, respectively). NX210c effect was sustained after 72-h treatment (Fig. 1B: -55.4%, -52.9% and − 50.0% for NX210c at 1, 10 and 100 µM compared with control; *p* = 0.0002, *p* = 0.0003 and *p* = 0.0004, respectively). Using the same experimental paradigm, we measured the TEER of endothelial cell monolayers by putting one electrode in the apical chamber containing endothelial cells and another one in the basolateral chamber using a resistance meter. The average TEER values of the control condition were 33 ± 3 Ω.cm^2^ and 31 ± 9 Ω.cm^2^ at 24 h and 72 h, respectively. Interestingly, NX210c also increased TEER of monolayers in a dose-dependent manner after both 24-h (Fig. 1C: +25.6% and + 28.1% for NX210c at 10 and 100 µM compared with control; *p* = 0.0002 and *p* < 0.0001, respectively) and 72-h treatments (Fig. 1D: +28.8% and + 39.6% for NX210c at 10 and 100 µM compared with control; *p* = 0.0001 and *p* < 0.0001, respectively). No effect of NX210c on TEER was observed at the lowest dose (i.e., 1 µM) compared with control (Fig. 1C-D: *p* = 0.0844 and *p* > 0.9999 after 24-h and 72-h treatments).

**Fig. 2:**
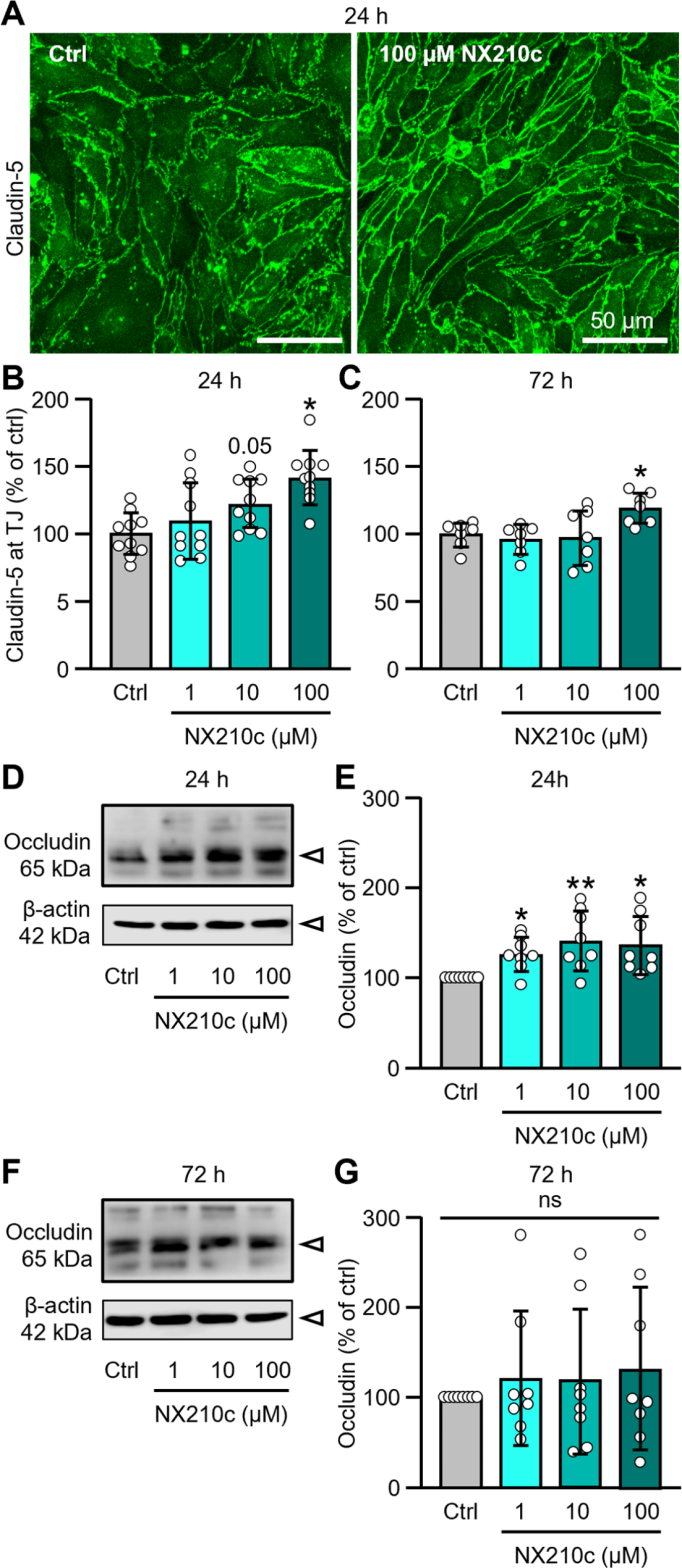
NX210c increases claudin-5 and occludin levels in bEnd3 mouse brain endothelial cell monolayers. (**A-C**) Monolayers of bEnd.3 mouse brain endothelial cells grown were treated with NX210c (1, 10, 100 µM) or its vehicle (cell culture water; ctrl) for 24–72 h, and then fixed and stained for claudin-5. (**A**) Representative photomicrographs of endothelial cells immunostained with claudin-5 (scale bar = 50 μm). (**B-C**) Quantification of claudin-5 immunoreactive area after 24-h (**B**) and 72-h treatments (**C**). One-way ANOVA followed by Dunnett’s multiple comparisons test: ^*^p < 0.05 compared with the control group, *n* = 10/group from two independent experiments (**B**; i.e., *n* = 6 biological replicates/group from experiment 1 and *n* = 4 biological replicates/group from experiment 2) and *n* = 7/group from two independent experiments (**C;** i.e., *n* = 4 biological replicates/group from experiment 1 and *n* = 3 biological replicates/group from experiment 2). (**D-G**) In parallel cultures, protein lysates were harvested after 24-h and 72-h treatments to perform western-blots. (**D**, **F**) Representative western-blots against occludin after 24-h (**D**) and 72-h treatments (**F**). (**E**, **G**) Corresponding quantification of occludin protein levels after 24-h (**E**) and 72-h treatments (**G**). Kruskal-Wallis followed by Dunn’s multiple comparisons test: ^**^p < 0.001, ^*^p < 0.05 compared with the control group, *n* = 8/group from three independent experiments (i.e., for each timepoint: *n* = 3 biological replicates/group from experiment 1, *n* = 2 biological replicates/group from experiment 2, and *n* = 3 biological replicates/group from experiment 3)

We have then evaluated the effect of NX210c on the presence of claudin-5 (known to be the most enriched TJ protein at the BBB) between endothelial cells by immunocytochemistry after 24 h and 72 h treatment with vehicle or NX210c at 1, 10 or 100 µM. Further, we have also evaluated the expression of claudin-5, occludin and ZO-1 by western-blot for protein levels and by RT-qPCR for mRNA levels. For that purpose, monolayers of bEnd.3 cells were treated with vehicle or NX210c, and mRNAs and proteins were extracted from cell layers 1 h, 3 h, 6 h, 24 h, 48 h and 72 h later. Interestingly, using immunocytochemistry, we have shown that NX210c at 100 µM induced a sustained increase in claudin-5 immunoreactive area at tight junctions (Fig. 2A-C: +42.5% and + 19.1% after 24-h and 72-h treatments; *p* = 0.0002 and *p* = 0.0416, respectively). Except a non-significant trend towards an increase in claudin-5 immunoreactive area with NX210c at 10 µM after 24-h treatment, no effect of NX210c at the 2 lowest doses (i.e., 1 and 10 µM) was observed on claudin-5 immunoreactive area after 24-h and 72-h treatments (Fig. 2A-C: *p* = 0.5512 and *p* = 0.0543 after 24-h treatment, and *p* = 0.9040 and *p* = 0.9809 after 72-h treatment for NX210c at 1 and 10 µM compared with control, respectively). In addition, a transient increase in protein levels was observed for occludin (Fig. 2D-E: +11.9%, + 14.6% and + 13.5% for NX210c at 1, 10 and 100 µM compared with control; *p* = 0.0309, *p* = 0.0053 and *p* = 0.0112, respectively); no effect of NX210c was observed on occludin protein levels with longer exposures (i.e., 48 h and 72 h; Supplementary Fig. 1, and Fig. 2F-G: Kruskal-Wallis, *p* = 0.9940), neither with shorter exposures (i.e., 1 h, 3 h and 6 h; Supplementary Fig. 1). No effect of NX210c was observed at all timepoints studied for claudin-5 and ZO-1 (Supplementary Fig. 1) protein levels measured by western-blot. Claudin-5, ZO-1 and occludin mRNA levels were similar in endothelial cell monolayers treated with vehicle or NX210c, regardless of the concentrations and times of exposure used (Supplementary Fig. 2), suggesting that NX210c had no effect on the transcriptional regulation of the expression of TJ proteins.

In summary, sustained increase in TEER and decrease in permeability to a 40-kDa FITC-dextran were observed when endothelial cells monolayers were treated with NX210c. Although no modification of claudin-5 mRNA and protein expression was observed (RT-qPCR, western-blot), 24-h and 72-h treatments with NX210c at 100 µM increased the presence of claudin-5 between endothelial cells (immunocytochemistry). In addition to claudin-5 redistribution at the endothelial surface, NX210c also increased the protein levels of occludin after 24 h of treatment whatever the dose. Overall, it is likely that (i) NX210c increases the levels of claudin-5 at tight junctions by promoting its redistribution at the surface of endothelial cells since no modification of its expression was found at the total mRNA and protein levels, and that (ii) the increased levels of claudin-5 at tight junctions, and to some extent occludin, lead to the increase in BBB resistance and to the decrease in BBB permeability observed in presence of NX210c.

### NX210c strengthens BBB integrity in static and dynamic models composed of human primary endothelial cells, astrocytes and pericytes

**Fig. 3:**
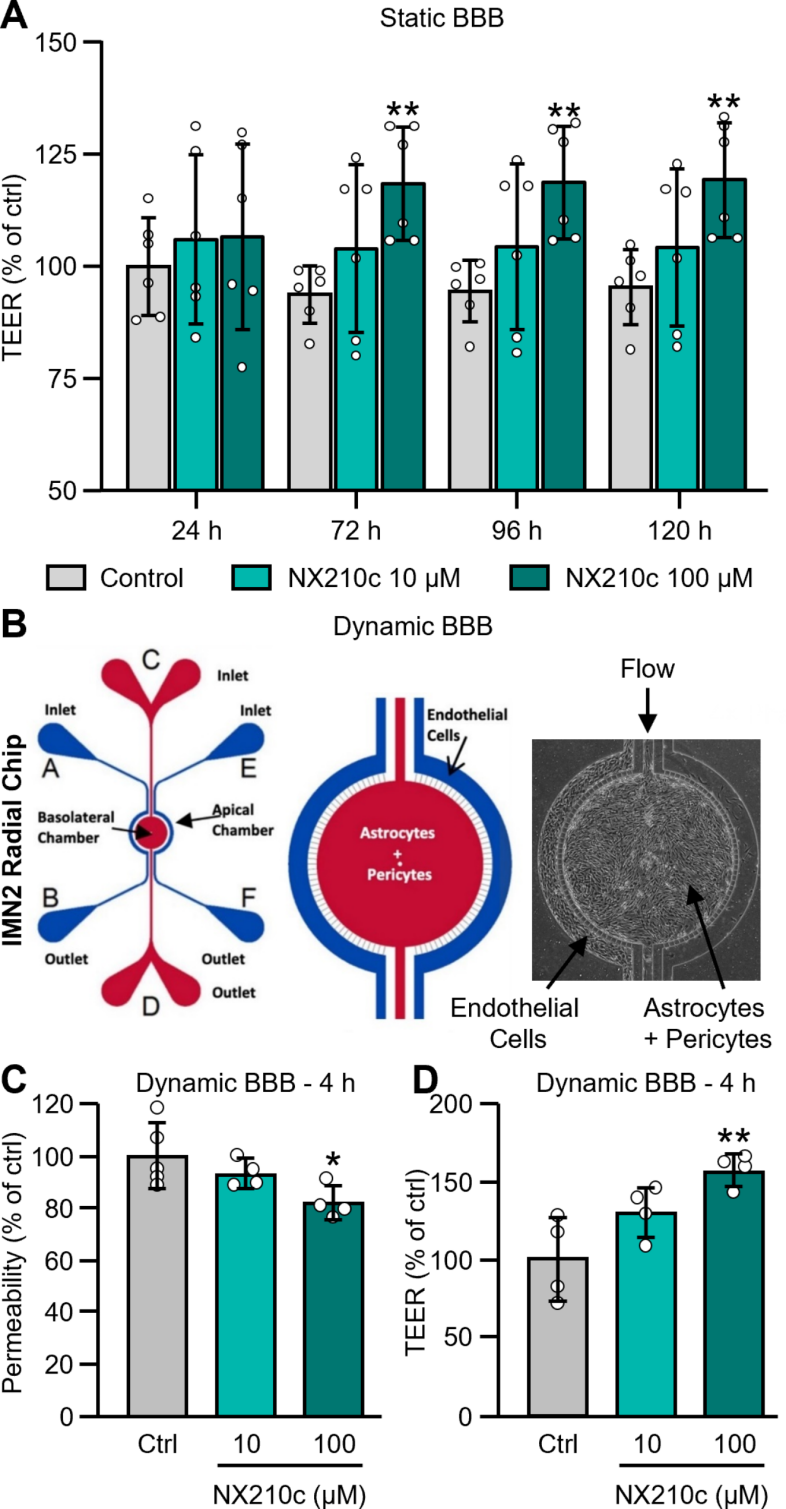
NX210c strengthens static and dynamic human BBB in vitro. (**A**) The TEER of a static BBB, composed by human primary endothelial cells (HBMVECs) in the apical chamber and of human primary astrocytes and pericytes in the basolateral chamber of transwell^®^ inserts, was followed up over time (24 h, 72 h, 96 h, 120 h) after exposure to NX210c (10, 100 µM) or its vehicle (cell culture water; ctrl). Two-way ANOVA followed by Dunnett’s multiple comparisons test: ^**^p < 0.01 compared with the control group, *n* = 6 transwell^®^ inserts/group from two independent experiments (i.e., *n* = 3 biological replicates/group from experiment 1 and *n* = 3 biological replicates/group from experiment 2). (**B-C**) The permeability of a dynamic BBB, composed by HBMVECs in the apical chamber and of human primary astrocytes and pericytes in the basolateral chamber of microfluidic chips, to a 4-kDa FITC-dextran was measured after exposure to NX210c (10, 100 µM) or its vehicle (cell culture water; ctrl) for 4 h. One-way ANOVA followed by Dunnett’s multiple comparisons test: ^*^p < 0.05 compared with the control group, *n* = 4–5 chips/group from one experiment. (**D**) In parallel chips, the TEER was measured using the same experimental design after 4-h treatment. One-way ANOVA followed by Dunnett’s multiple comparisons test: ^**^p < 0.01 compared with the control group, *n* = 4 chips/group from one experiment

We have evaluated the TEER of HBMVECs in presence of human primary pericytes and astrocytes in transwell^®^ inserts (static BBB) using 2 different concentrations of NX210c (10 and 100 µM) or its vehicle (cell culture water) over time (24 h, 72 h, 96 h, 120 h). The average TEER value of the control condition was 166 ± 19 Ω.cm^2^ at 24 h. As observed with mouse endothelial cell monolayers, NX210c at 100 µM also significantly increased TEER from 72 h after the treatment and up to at least 5 days of treatment (Fig. 3A: +24.6%, + 24.3% and + 23.9% after 72-h, 96-h and 120-h treatments; *p* = 0.0061, *p* = 0.0062, *p* = 0.0079, respectively). No effect of NX210c on TEER was observed at 100 µM compared with control after 24 h of treatment (Fig. 3A: *p* = 0.7207). No effect of NX210c on TEER was observed at 10 µM compared with control at any timepoint (Fig. 3A: *p* = 0.7333, *p* = 0.3977, *p* = 0.4093 and *p* = 0.4718 after 24-h, 72-h, 96-h and 120-h treatments).

We have then evaluated the permeability of HBMVECs in presence of human primary pericytes and astrocytes in microfluidic devices (dynamic BBB) to a 4-kDa FITC-dextran using 2 different concentrations of NX210c (10 and 100 µM) or its vehicle (cell culture water) (Fig. 3B). The FITC-dextran was placed on the apical chamber containing endothelial cells, and the fluorescence was measured 1 h later on the basolateral chamber. This model recapitulates shear stress induced by the blood into the vessels and is therefore physiologically more relevant than static models using transwell^®^ inserts. As observed in the 2 other models, NX210c also reduced permeability to a 4-kDa FITC-Dextran (Fig. 3C: -18.0% compared with control; *p* = 0.0259), meanwhile increasing TEER at 100 µM 4 h after the treatment (Fig. 3D: +57.2% compared with control; *p* = 0.0041), in this human 3D dynamic BBB model exhibiting shear stress; the average TEER value of the control condition was 28 ± 7 kΩ at 4 h (it corresponds to 4.82 in Ω.cm^2^ because the surface area of the barrier in the chip is 0.000153 cm^2^. We do not present data as a function of the surface area in a chip because of the size difference compared to the much higher surface area in a traditional transwell^®^, as well as differences in the electrode placements). No significant effect of NX210c at 10 µM on the permeability to the 4-kDa FITC-dextran and on TEER was observed compared with control (Fig. 3C-D: *p* = 0.4877 and *p* = 0.0898, respectively).

In summary, increase in TEER and/or decrease in permeability to a FITC-dextran were observed when static or dynamic human BBBs were treated with NX210c.

### Repeated intraperitoneal injections of NX210c increase claudin-5 and occludin levels in the brain of old mice

**Fig. 4:**
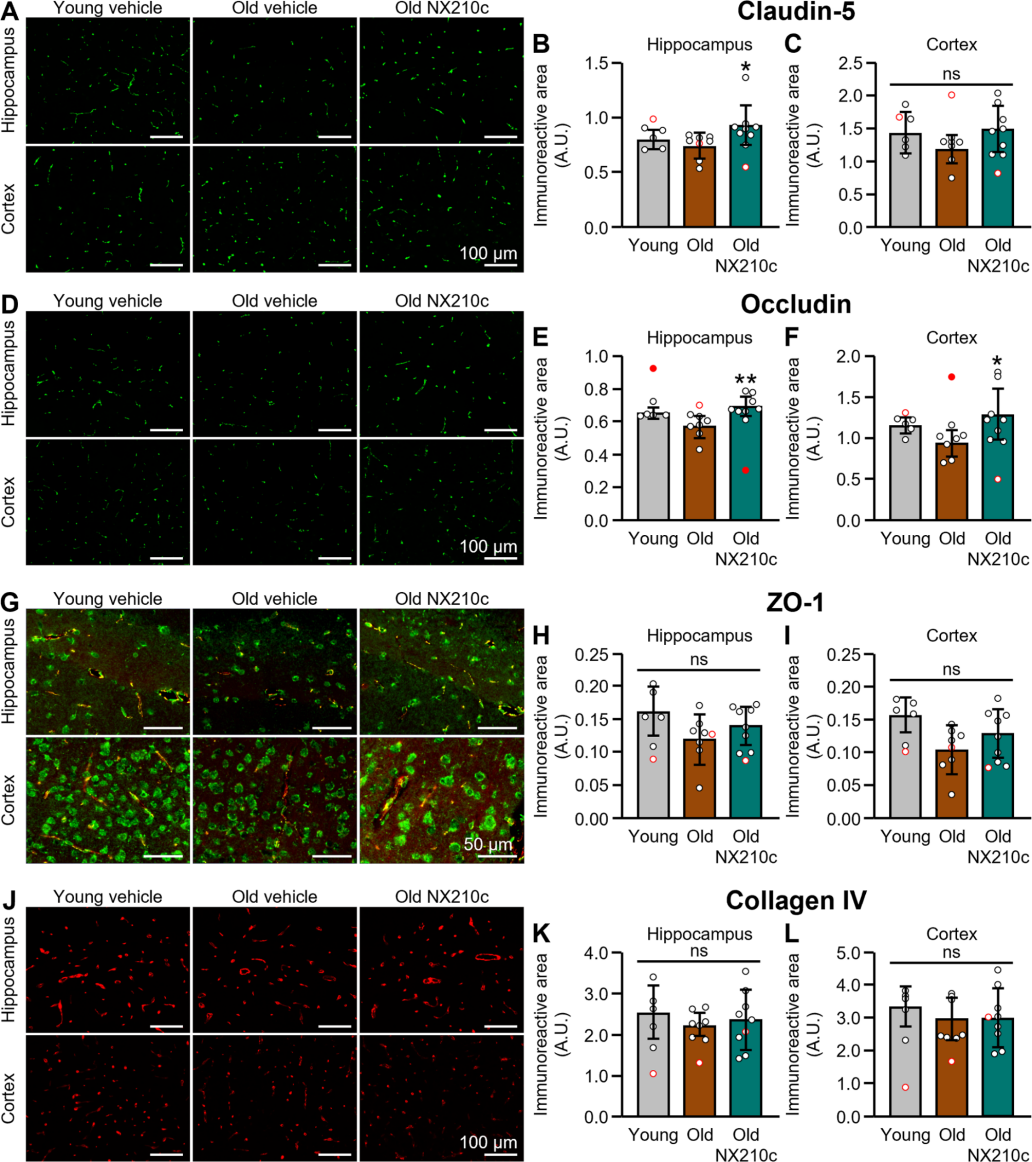
Repeated systemic administrations of NX210c increase claudin-5 and occludin levels in the brain of 21-month-old mice. (**A**, **D**, **G**, **J**) Representative photomicrographs of claudin-5 (**A**), occludin (**D**), ZO-1 (green) / claudin-5 (red) (**G**), and collagen IV (**J**) in the hippocampus (top) and the cortex (bottom) of young (3-month-old) and old (21-month-old) mice treated or not with NX210c at 10 mg/kg or its vehicle (water for injection) intraperitoneally for 5 days, once a day, and sacrificed one day after the last injection. Scale bars: 50–100 μm. (**B-C**) Quantifications of claudin-5 immunoreactive area in the hippocampus (**B**) and the cortex (**C**), expressed as a percentage of the young vehicle group. Kruskal-Wallis followed by Dunn’s multiple comparisons test: ^*^p < 0.05 old vehicle group vs. old NX210c group (**B**), and Kruskal-Wallis: *p* > 0.05 (**C**). (**E-F**) Quantifications of occludin immunoreactive area in the hippocampus (**E**) and the cortex (**F**), expressed as a percentage of the young vehicle group. Kruskal-Wallis followed by Dunn’s multiple comparisons test (**E**) or one-way ANOVA followed by Dunnett’s multiple comparisons test (**F**): ^**^p < 0.01, ^*^p < 0.05 old vehicle group vs. old NX210c group. (**H-I**) Quantifications of ZO-1 immunoreactive area in the hippocampus (**H**) and the cortex (**I**), expressed as a percentage of the young vehicle group. One-way ANOVA: *p* > 0.05. (**K-L**) Quantifications of collagen IV immunoreactive area in the hippocampus (**K**) and the cortex (**L**), expressed as a percentage of the young vehicle group. One-way ANOVA (**K**) or Kruskal-Wallis (**L**): *p* > 0.05. *n* = 5 vehicle-treated young mice, *n* = 7 vehicle-treated old mice, and *n* = 8 NX210c-treated old mice. Significant outliers are identified in red full circles (**E-F**); these outliers were removed from other analyses for consistency, and are identified as red empty circles

To evaluate the potential of NX210c to modulate tight junction protein levels in vivo, we used old mice as a first proof of concept; indeed, in the mouse brain, old age is associated with loss of tight junction proteins accompanied by an increase in the permeability of the BBB [50–57]. The dose, frequency, and administration route used in this study were selected based on prior proofs of concept of NX210c efficacy in several neurological disorders to promote functional recovery (unpublished data). Importantly, systemic administrations of the peptide at the same dose were shown to be safe and well tolerated in single [58] and multiple ascending dose (unpublished data) clinical studies. Regarding the treatment duration chosen in this study, it was an assumption from our in vitro BBB results showing that NX210c quickly strengthens the BBB (Fig. 3C) and that its effect is durably maintained overtime (Figs. 1B and D and 3A). A non-significant decrease in claudin-5 immunoreactive area was observed in the hippocampus of 21-month-old male mice compared with that of 3-month-old mice (Fig. 4A, B: -7.7% in vehicle-treated old mice compared with vehicle-treated young mice, *p* = 0.9665). Interestingly, NX210c at 10 mg/kg given i.p. once a day for only 5 days to old mice increased claudin-5 immunoreactive area in the hippocampus (Fig. 4A, B: +24.1% in NX210c-treated old mice compared with vehicle-treated old mice, *p* = 0.0134). No significant modification of claudin-5 immunoreactive area was found between groups in the cortex (Fig. 4A, C: Kruskal-Wallis, *p* = 0.2540).

A non-significant decrease in occludin immunoreactive area was also observed during aging in the hippocampus (Fig. 4D, E: -13.2% in vehicle-treated old mice compared with vehicle-treated young mice, *p* = 0.1072) and the cortex (Fig. 4D, F: -18.2% in old vehicle mice compared with young mice, *p* = 0.2439). Interestingly, NX210c treatment significantly increased occludin immunoreactive area both in the hippocampus (Fig. 4D, E: +19.2% in NX210c-treated old mice compared with vehicle-treated old mice, *p* = 0.0016) and the cortex (Fig. 4D, F: +30.6% in NX210c-treated old mice compared with vehicle-treated old mice, *p* = 0.0178).

No modification of ZO-1 immunoreactive area was found between groups in the hippocampus (Fig. 4G, H: One-way ANOVA, *p* = 0.1489) and in the cortex (Fig. 4G, I: One-way ANOVA, *p* = 0.0586).

Importantly, no modification of collagen IV immunoreactive area was found between groups in the hippocampus (Fig. 4J, K: One-way ANOVA, *p* = 0.7257) and in the cortex (Fig. 4J, L: Kruskal-Wallis, *p* = 0.7119), suggesting a similar number of vessels between groups.

In summary, NX210c given i.p. once a day for only 5 days increased the cerebral levels of the tight junction proteins claudin-5 and occludin in old mice.

## Discussion

Using several in vitro BBB models in three different labs, we have shown that NX210c peptide increased the expression of TJ proteins, claudin-5 and occludin, between endothelial cells, and favorably modulated the integrity and permeability of the BBB, including in a primary human dynamic model exhibiting endothelial cells, astrocytes and pericytes. In an in vivo proof of concept of NX210c effect on the BBB, intraperitoneal injections of the peptide once a day for 5 days to old mice increased the protein expression of claudin-5 and occludin in the hippocampus, and in the cortex too for occludin.

The brain vasculature is characterized by continuous TJ complexes between endothelial cells which allows BBB tightness and restricts passage of immune cells and molecules between the blood and the brain, and ensures a proper functioning of the brain [59]. Claudin-5, occludin and ZO-1 are closely intertwined to form TJs that ensure the integrity of the BBB and control paracellular permeability; claudin-5 and occludin are transmembrane proteins of endothelial cells, whereas ZO-1 present inside endothelial cells ensures the structural support of the TJs. In various neurodegenerative diseases and injuries to the CNS, TJs are degraded which readily interrupts the BBB that leaks blood-borne cells and proteins. Elevated BBB leakage is associated with:

- greater psychiatric morbidity in bipolar patients [7];
- higher disease severity and clinical disabilities in patients with neuronal surface antibody-associated autoimmune encephalitis [24, 26], neuromyelitis optica spectrum disorder [29, 31] and ALS [11, 60];
- higher disease severity and cognitive function deterioration in acquired brain injury conditions such as stroke [61] and traumatic brain injury (TBI) [25, 27, 30] and mild cognitive impairment individuals [32].

BBB integrity is also compromised during normal aging [51–53]. For example, a decreased coverage of vessels with claudin-5 was observed by immunofluorescence in the brain of 16-22-month-old C57B/6J mixed-gender mice, and accordingly a ∼ 30% decrease in protein levels of claudin-5 in lysates of isolated brain vessels was measured by western-blot compared with 2-6-month-old littermates [50]. A strong reduction in claudin-5 protein and mRNA levels was also observed by another group in the hippocampus of 24-month-old C57BL/6J male mice compared with 10-month-old littermates [54]. Interestingly, positive correlation between the extravasation of a FITC-inulin into the brain and age was found in mice [50]. Furthermore, the presence of micro-hemorrhages was detected in the thalamus of 17–18 and 24-25-month-old C57B/6J male mice, and was associated with sensorimotor and memory deficits [56]. Stamatovic’s group also showed a huge decrease in claudin-5 immunofluorescence with age in post-mortem human brains, that was associated with an increased extravasation of the blood component fibrinogen [50]. Accordingly, other groups have reported an enhanced volume transfer constant (K_trans_) of gadolinium by dynamic contrast-enhanced magnetic resonance imaging (DCE-MRI), reflecting an enhanced BBB leakage, in the elderly brain including the cortex and hippocampus, in both white and grey matters [56, 57]. As mentioned before for TBI and stroke, BBB leakage is closely linked to cognitive dysfunction in aging too [28, 62]. In our study, we chose to use 21-month-old mice based on the literature showing an increased permeability of the BBB associated with decreased protein levels of tight junction proteins in 18-21-month-old mice [50, 63]. The older the mice, the higher the risk of death, therefore 21-month-old of age was a good compromise to see BBB dysfunction whilst not having a massive loss of mice due to advanced age. Unfortunately, we measured a moderate non-significant loss of TJ proteins, claudin-5 (∼ 10–20%), occludin (∼ 15–20%) and ZO-1 (∼ 25–35%), in the cortex and hippocampus of 21-month-old C57B/6J male mice compared with 3-month-old littermates. It might be attributed to a lower number of animals in the control group (*n* = 5) compared with other groups (*n* = 6–7), the age of mice (not old enough? ), or the method used to measure the levels of tight junctions (more accurate in terms of specificity than western-blot but not in terms of precision). In this study, we have not evaluated the effect of NX210c on tight junction proteins in young mice, but, interestingly, systemic injections of NX210c once daily for 5 days significantly increased levels of claudin-5 and occludin in the brain of old mice. It would be interesting in future studies to determine if NX210c strengthens BBB per se or counteracts specific aberrant processes triggered during aging such as the immune response [64]. Nevertheless, in the in vitro models used in this study, endothelial cells were not challenged with inflammatory cytokines or immune cells, and yet NX210c increased BBB integrity.

In vitro, we have shown that NX210c reduced the permeability of a 40-kDa FITC-dextran by about 60% after 24-h and 72-h treatments regardless of the dose used (i.e., 1, 10 or 100 µM) compared with the control condition in mouse bEnd.3 endothelial cell monolayers. On the contrary, a dose- and time-dependent increase in TEER was observed in the same conditions at 10 and 100 µM, but no effect was observed for this parameter at 1 µM. We acknowledge discrepancies between the amplitude of TEER and dextran permeability, and between the dose efficacy in each of these parameters; in the literature, both linear and non-linear relationships between TEER and permeability parameters can be observed [65, 66]. However, the reduction of dextran permeability after 24 h of treatment is in line with the increase in occludin protein levels observed at the same timepoint regardless of the dose used. Similarly, claudin-5 immunoreactive area was increased in a dose-dependent manner after 24 h of treatment as observed for TEER with no effect detected for the 1-µM condition. The only TJ protein whose expression was still elevated after 72 h of treatment and only at 100 µM was claudin-5; arguably, it could be possible that NX210c modulates other components that strengthen endothelial cell monolayers to explain the sustained decrease in dextran permeability at 1 and 10 µM in this model. Importantly, we have shown that NX210c also increased BBB integrity using more complex in vitro models (tricultures of human endothelial cells, pericytes and astrocytes in static and dynamic conditions) despite some differences. For instance, in the static human BBB model, NX210c at 100 µM was ineffective at modulating TEER after 24 h of treatment, and NX210c at 10 µM did not modulate this parameter at any timepoint. Noteworthy, TEER in this model was about 5 times higher in the control condition than that of mouse bEnd.3 endothelial cell monolayers, likely due to the presence of astrocytes and pericytes that tighten the BBB. It might be that it is more difficult for NX210c (and any compound for that matter) to strengthen a tighter BBB (i.e., it might take more time and a higher dose to achieve its effects). In the dynamic human BBB model, a 4-kDa FITC-dextran was used, in comparison to the 40-kDa FITC-dextran used in mouse bEnd.3 endothelial cell monolayers [45] due to the shear stress forces applied inducing a different development of the BBB. Furthermore, the duration of treatment in this model due to shear stress endured by endothelial cells cannot be as long as for static models, therefore the supplier (SynVivo) recommends shorter treatments (i.e., 4 h).

Maintaining or restoring BBB integrity may represent a disease-modifying target to halt the progression of several neurological disorders, yet only few therapeutic strategies are developed at preclinical and clinical stages. Overall, two main types of strategies are employed: (1) restoring normal levels of TJ proteins, or (2) reducing leukocyte infiltration into the brain parenchyma (= diapedesis). In addition, one may consider strategies counteracting blood-derived components that have leaked into the brain parenchyma to reduce neuroinflammation and neurodegeneration. Interestingly, BBB integrity in MS patients receiving natalizumab (Biogen, Cambridge, MA, USA) is currently evaluated in a phase IV clinical trial although the effect on BBB integrity was not originally claimed. Most of the companies with BBB repair agents are at discovery or early preclinical stage, while NX210c was recently shown to be safe and well tolerated in a phase Ib multiple ascending dose study (unpublished data). Furthermore, in addition to repairing the BBB, NX210c is also protective to neurons, both being important to the functional outcome of patients suffering from neurological disorders. Importantly, given the short size of the peptide (only 12 natural amino acids), the risk to develop anti-drug antibodies (ADA) is considered very low. Accordingly, pharmacokinetics parameters were not modified in preclinical and clinical studies, even after repeated administrations, excluding the presence of ADA.

Mechanistically, we hypothesize that NX210c mediates BBB strengthening by acting on integrins containing the β_1_-subunit. Indeed, we have previously shown that NX210c lost its neuroprotective effect on rat neurons exposed to glutamate-induced excitotoxicity when they were pre-treated with an antibody targeting β_1_-integrins [35]; similarly, we have recently shown that the increase in N-methyl-D-aspartate (NMDA) receptor post-synaptic currents induced by NX210c [36] was mediated by the same receptors (unpublished data). NX210c acting on β_1_-integrins to strengthen BBB integrity would be in line with several groups showing that β_1_-integrins are essential for stabilizing the BBB and ensuring BBB integrity [67–70]. Indeed, primary cerebral endothelial cells exposed to a monoclonal antibody targeting β_1_-integrin displayed an increased BBB permeability to 40 and 150-kDa FITC-dextrans and reduced TEER and claudin-5 coverage [68]. Accordingly, when injected into the striatum of C57BL/6 male mice, the anti-β_1_-integrin antibody induced a remarkable immunoglobulin G (IgG) extravasation after 24 h [68]. Similarly, repeated daily intraperitoneal injections of a β_1_-integrin function-blocking antibody greatly increased hypoxia-induced BBB leaks of fibrinogen, notably in the cortex, striatum and corpus callosum, in both 2-month-old (young) and 20-month-old C57BL6/J female mice [69]. The same group further described that similar exposure to this β_1_-integrin antibody to 2-month-old C57BL6/J female mice also enhanced blood-spinal cord barrier permeability to fibrinogen in normoxic and hypoxic conditions, which lead to microglia activation and demyelination [70]. To prove that NX210c effect on BBB integrity is mediated by β_1_-integrins, the challenge will be to find a suitable concentration of an anti-β_1_-integrin antibody that would not ensure BBB dysfunction by itself whilst blocking NX210c effect on tight junction protein levels and BBB permeability.

For decades, the primary focus of drug development in the CNS field has been to save neurons [71], yet it may be of significant importance for future therapeutic strategies to rescue BBB integrity to prevent neurodegenerative processes and subsequent brain damages [72–77]. By promoting neurovascular health, NX210c provides a therapeutic opportunity to reduce BBB leakage which may be disease-modifying in various neurological disorders with high unmet needs for which the BBB is impaired. Among a broad range of potential applications, ALS has been selected to reach the clinical demonstration of the benefit of NX210c as a neurovascular unit/BBB repair treatment; a phase II clinical trial is expected to be launched in 2024 (NCT06365216).

## Electronic supplementary material

Below is the link to the electronic supplementary material.

Supplementary Material 1

## Data Availability

The data that support the findings of this study are available from the corresponding author, upon reasonable request.

## Abbreviations

AD: Alzheimer’s disease
ADA: Anti-drug antibodies
ALS: Amyotrophic lateral sclerosis
ANOVA: Analysis of variance
BBB: Blood-brain barrier
bEnd.3: Mouse brain endothelial cell line
CNS: Central nervous system
Ctrl: Control
D: Day
Da: Dalton
DCE-MRI: Dynamic contrast-enhanced magnetic resonance imaging
DMEM: Dulbecco’s modified eagle’s medium
DPBS: Dulbecco’s phosphate-buffered saline
EBM: Endothelial basal medium
EC: Endothelial cell
ECGM: Endothelial cell growth medium
ERS: Electrical resistance system
FBS: Fetal bovine serum
FITC: Fluorescein isothiocyanate
HBMVEC: Human brain microvascular endothelial cells
HBVP: Human brain vascular pericytes
HEPES: 4-(2-Hydroxyethyl)-1-piperazine ethanesulfonic acid
IgG: Immunoglobulin G
i.p.: Intraperitoneally
K_trans_: Volume transfer constant
mRNA: Messenger ribonucleic acid
mTor: Mammalian target of rapamycin
NEAA: Non-essential amino acids
NHA: Normal human astrocytes
NMDA: N-methyl-D-aspartate
P/S: Penicillin/streptomycin
Papp: Apparent permeability coefficient
PBS: Phosphate buffered saline
PD: Parkinson’s disease
PFA: Paraformaldehyde
PI3K: Phosphoinoside-3-kinase
ROI: Region of interest
RT: Room temperature
RT-qPCR: Reverse transcription quantitative polymerase chain reaction
SCO: Subcommissural organ
SD: Standard deviation
SDS: Sodium dodecyl sulfate
T/E: Trypsin/ethylenediaminetetraacetic acid
TBI: Traumatic brain injury
TBS: Tris-buffered saline
TEER: Transendothelial electrical resistance
TJ: Tight junction
TSR1: Type 1 thrombospondin repeats
ZO-1: Zonula occludens-1
