# Supplementary material for: NX210c drug candidate peptide strengthens mouse and human blood-brain barriers
Source: Fluids Barriers CNS. 2024 Sep 27;21:76. doi: 10.1186/s12987-024-00577-x (PMC11438064; doi:10.1186/s12987-024-00577-x)
Supplement: Supplementary file 1 — Supplementary Material 1 [file 12987_2024_577_MOESM1_ESM.docx]

**Supplementary materials**

**Supplementary Methods**

*Reverse transcription quantitative polymerase chain reaction (RT-qPCR)*

bEnd3 cells seeding and treatment with NX210c or its vehicle were performed as described in the main manuscript for collection of protein lysates for western-blots. Messenger ribonucleic acids (mRNAs) were isolated using E.Z.N.A Total RNA Kit I (R6834-02, Omega Bio-tek, Norcross, Georgia) and complementary deoxyribonucleic acids (cDNAs) were reverse-transcribed from 1 µg of mRNAs using the high-capacity cDNA Reverse transcription Kit (4368814, Applied Biosystems, Foster City, CA, USA). cDNAs were diluted 1:10 with nuclease free water (10977035, Biosciences, Dublin, Ireland) and RT-qPCR was performed on a StepOnePlus machine (Applied Biosystems) with SensiFAST^TM^ SYBR® Hi-ROX Kit (BIO-92020, Bioline, London, UK) as a fluorescent dye. RT-qPCR conditions were as follows: (95 °C × 10 s, 60 °C × 30 s) × 40 cycles, 95 °C × 15 s, 60 °C × 1 min, 95 °C × 15 s, 60 °C × 15 s. Primer sequences (Sigma-Aldrich, Saint-Louis, MO, USA) were as follows [1]: claudin-5 forward 5’-TTTCTTCTATGCGCAGTTGG-3’ and reverse 3’-GCAGTTTGGTGCCTACTTCA-5’, occludin forward 5’-ACAGTCCAATGGCCTACTCC-3’ and reverse 3’-ACTTCAGGCACCAGAGGTGT-5’, ZO-1 forward 5’-CCACCTCTGTCCAGCTCTTC-3’ and reverse 3’-CACCGGAGTGATGGTTTTCT-5’, β-actin forward 5’-GGGAAATCGTGCGTGACAT-3’ and reverse 3’-GTGATGACCTGGCCGTCAG-5’. Relative target transcript levels were normalized to those of the house keeping gene β-actin using the comparative C_T_ method (∆∆C_T_). The results are expressed as percentages of the control group.

**Supplementary Figures**

**
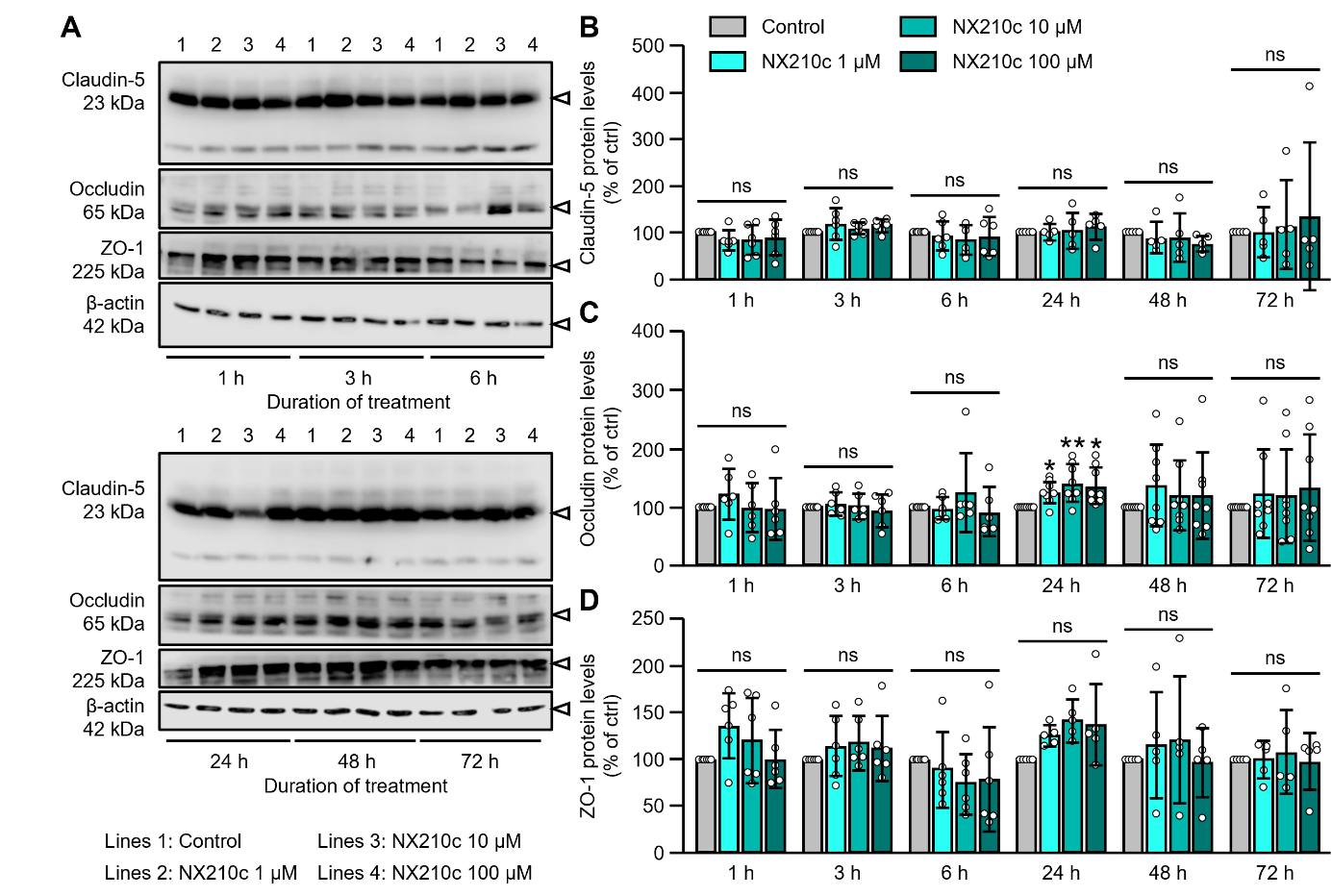
**

**Supplementary Fig. 1** NX210c modulates the protein levels of occludin, but not claudin-5 and ZO-1, in mouse brain endothelial cells *in vitro*. Mouse brain endothelial bEnd.3 cells were exposed to vehicle or different doses of NX210c (1, 10, 100 µM) for 1 h, 3 h, 24 h, 48 h or 72 h, and the cell layers collected for western-blots (**A**) to quantify the protein levels of claudin-5 (**B**), occludin (**C**) and ZO-1 (**D**). The data are expressed as percentage of the control group. For each timepoint, a Kruskal-Wallis followed by Dunn’s multiple comparisons tests was performed: ^**^p < 0.01, ^*^p < 0.05 NX210c groups *vs* control group after 24-h treatment (occludin), otherwise p > 0.05, n=5-8/group (ie, for each tight junction protein after 1-h, 3-h and 6-h treatments: n=6/group from two independent experiments (ie, n=3 biological replicates/group from experiment 1 and n=3 biological replicates/group from experiment 2); for claudin-5 and ZO-1 after 24-h, 48-h and 72-h treatments: n=5/group from two independent experiments (ie, n=3 biological replicates/group from experiment 1 and n=2 biological replicates/group from experiment 2); for occludin after 24-h, 48-h and 72-h treatments: n=8/group from three independent experiments (ie, n=3 biological replicates/group from experiment 1, n=2 biological replicates/group from experiment 2, and n=3 biological replicates/group from experiment 3)).

**
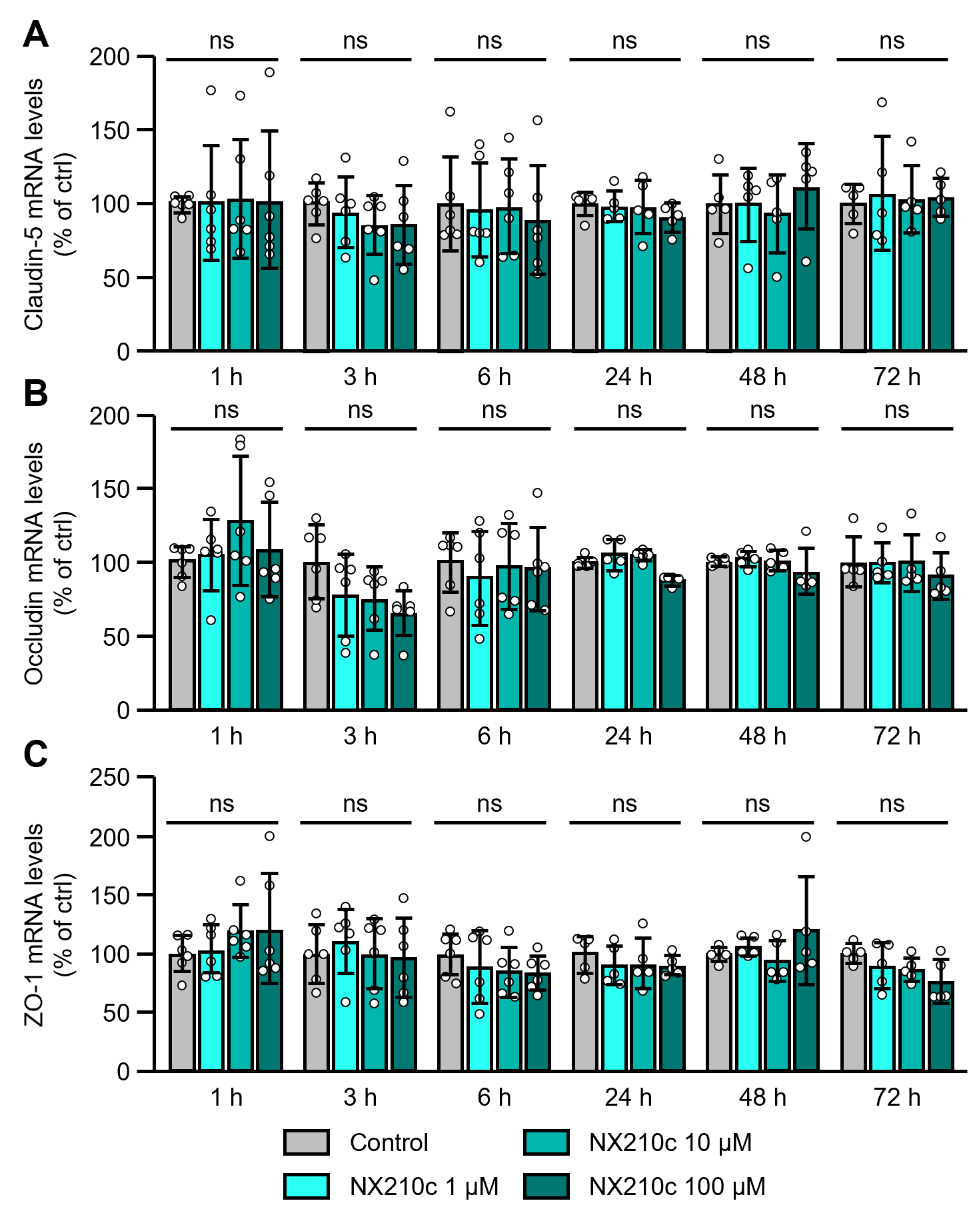
**

**Supplementary Fig. 2** NX210c does not modulate the transcriptional regulation of the expression of claudin-5, occludin and ZO-1 in mouse brain endothelial cells *in vitro*. Mouse brain endothelial bEnd.3 cells were exposed to vehicle or different doses of NX210c (1, 10, 100 µM) for 1 h, 3 h, 24 h, 48 h or 72 h, and the cell layers collected for RT-qPCR to quantify mRNA expression of claudin-5 (**A**), occludin (**B**) and ZO-1 (**C**) and. The data are expressed as percentage of the control group. For each timepoint, one-way ANOVA followed by Dunnett’s multiple comparisons tests or Kruskal-Wallis followed by Dunn’s multiple comparisons tests was performed based on the normality of the data and equality of the variances: p > 0.05, n=5-6/group (ie, for each tight junction protein after 1-h, 3-h and 6-h treatments: n=6/group from two independent experiments (ie, n=3 biological replicates/group from experiment 1 and n=3 biological replicates/group from experiment 2); for each tight junction protein after 24-h, 48-h and 72-h treatments: n=5/group from two independent experiments (ie, n=3 biological replicates/group from experiment 1 and n=2 biological replicates/group from experiment 2)).

**Supplementary References**

[1] Greene C, Hanley N, Reschke CR, Reddy A, Mäe MA, Connolly R, et al. Microvascular stabilization via blood-brain barrier regulation prevents seizure activity. Nat Commun. 2022;13(1):2003.
